# From pilot to policy: why AI health interventions fail to scale in developing countries

**DOI:** 10.3389/fdgth.2026.1699005

**Published:** 2026-01-29

**Authors:** Jeena Joseph

**Affiliations:** Department of Computer Applications, Marian College Kuttikkanam Autonomous, Kuttikkanam, Kerala, India

**Keywords:** artificial intelligence in healthcare, developing countries, pilotitis, health policy, digital health systems, scalability

## Introduction: the pilot paradox

Across developing countries, artificial intelligence (AI) in healthcare has become the subject of remarkable optimism. From tuberculosis detection algorithms to AI-powered maternal health monitoring, new tools are constantly being tested in hospitals, primary health centers, and community health programs (1, 2). Governments, multilateral organizations, and donors increasingly support such experiments, seeing AI as a potential shortcut to address chronic shortages of specialists, infrastructure gaps, and inefficiencies in service delivery (3, 4).

However, despite this proliferation of pilots, very few AI initiatives advance to nationwide deployment or long-term institutional adoption. This mismatch—high pilot activity but low policy integration—has been described as the “pilotitis” syndrome of digital health (5). Instead of transforming health systems, many AI projects become isolated, donor-driven, and unsustainable. Hospitals may run a pilot for a year with external support, but once funding ends, servers go offline, trained staff move on, and policymakers shift priorities (6). The technology itself may be promising, but the enabling ecosystem remains weak.

This paradox reveals a systemic problem. While researchers and start-ups focus on demonstrating algorithmic accuracy and efficiency gains, governments face the harder question: How can AI be responsibly and sustainably embedded into complex, resource-constrained health systems? Answering this question requires moving beyond narrow evaluations of technical feasibility toward a broader analysis of governance, trust, policy frameworks, and system readiness (1, 5).

This article critically reflects on why AI interventions remain stuck at the pilot stage in developing countries, despite their apparent promise. Drawing from case experiences in Brazil, India, and selected sub-Saharan African countries such as South Africa, Kenya, and Rwanda, it analyzes recurring barriers that hinder scaling. It then proposes a five-pillar roadmap for transforming successful pilots into sustainable policy-backed programs. The argument is simple: scaling AI in healthcare is less about proving algorithms work, and more about building health systems that can work with algorithms.

### Barriers to scaling AI in developing countries

The World Health Organization (WHO) identifies similar obstacles in its primary guidance on AI for health. The WHO Ethics and Governance of AI for Health report highlights challenges such as poor data quality, algorithmic bias, and unclear accountability, all of which make AI harder to scale than conventional digital health tools (7). The WHO Regulatory Considerations on AI for Health further stresses the need for documentation, transparency, and post-market monitoring—requirements that many pilots in developing countries fail to meet (8). WHO's recent guidance on large multimodal AI models adds that without explainability and local validation, AI systems risk unreliable performance in diverse populations (9). These primary sources reinforce that the scaling barriers seen in Brazil, India, and sub-Saharan African countries are part of a wider global pattern.

Although many of the structural constraints discussed in this paper are also relevant to digital health more broadly, AI introduces a distinct and more demanding layer of complexity. AI tools require large, high-quality datasets, reliable data pipelines, mechanisms for explainability, and continuous model updating—needs that far exceed those of routine telemedicine or electronic record systems. Challenges such as model drift, algorithmic bias, and unclear liability pathways make AI considerably more difficult to scale within low-resource health systems. Therefore, while digital health infrastructure forms the foundation, the barriers outlined below are amplified in the context of AI, which imposes stricter requirements for data governance, computational capacity, and institutional trust.

• Fragmented Digital Ecosystems: A major challenge lies in the lack of interoperability across health information systems. For instance, Brazil's Unified Health System (SUS) historically operated with multiple government and private electronic health record (EHR) platforms that did not communicate with one another. Only recently did collaborative initiatives begin integrating municipal and federal data platforms. Without standardized data structures, AI tools cannot operate seamlessly across facilities, leading to duplication and inefficiency (10, 11). Similarly, in several sub-Saharan African countries—particularly Kenya, South Africa, and Rwanda—vertical health programs (HIV, TB, maternal health) each deploy their own digital tools. AI pilots often integrate with one vertical system but not with the broader national architecture, limiting scalability (12, 13).
• Dependence on Donor Funding: AI pilots in the Global South are frequently initiated and sustained by donor or NGO funding, which prioritizes innovation but rarely guarantees long-term financing. Once grants end, governments often lack budgetary space to absorb the costs of licensing, cloud infrastructure, or technical support. This creates a cycle where pilots demonstrate feasibility but collapse due to financial unsustainability (14). For example, AI-based diagnostic imaging tools piloted in countries such as Kenya and Rwanda showed strong results for TB detection, but when donor funding ended, hospitals struggled to pay for recurring software subscriptions and data storage (15–17).
• Weak Policy and Regulatory Frameworks: Scaling AI requires clear governance structures—who is accountable when AI makes a diagnostic error? How should liability be shared between developers, clinicians, and hospitals? In many developing countries, such questions remain unresolved. Regulatory frameworks for AI in healthcare are either nascent or absent, creating uncertainty for policymakers and reluctance among clinicians to adopt AI systems (18, 19). Data protection laws also vary widely. While countries like Brazil have robust frameworks (LGPD), many others lack clear guidelines on data storage, sharing, and cross-border flows, raising ethical and legal concerns (20).
• Low Levels of Trust and Acceptance: Trust is critical for adoption. Yet, clinicians often perceive AI as a threat to professional autonomy, fearing it may replace rather than augment their expertise. Patients, on the other hand, may distrust algorithms trained on foreign datasets that do not reflect local populations. Without transparency and explainability, AI tools risk rejection, even when technically accurate. Evidence from the UK's NHS RADICAL study showed that explainability and clinician confidence were key to adoption. These concerns are magnified in resource-limited contexts where misdiagnosis can have severe consequences (21, 22).
• Inadequate Infrastructure: Successful AI deployment requires stable internet, reliable electricity, robust servers, and secure data storage. Many rural hospitals in developing countries lack these basics. Without such infrastructure, even the most sophisticated AI system cannot function reliably. As Byberg and Crimi note, hospitals often rush to adopt AI without addressing foundational gaps, leading to poor performance and wasted resources (23).
• Fragmented Political Commitments: Scaling is also constrained by federal governance structures, political turnover, and bureaucratic fragmentation. In countries where health responsibilities are shared across national and subnational governments, achieving consensus on digital strategies is challenging. For example, Brazil's data federation required political will across municipal and federal levels, without which integration would not have been possible (2, 14).

### Lessons from case studies

• Brazil: Progress in Interoperability: Brazil's recent experience integrating primary and hospital data systems demonstrates that political commitment, technical architecture, and governance alignment are critical for scaling. By establishing data federations across municipal and federal levels, Brazil created a foundation for AI applications in predictive analytics and care coordination. However, the process was slow, resource-intensive, and dependent on continuous political support (24).
• India: Mixed Record in Digital Health: India's ambitious push for digital health, through initiatives like Ayushman Bharat Digital Mission (ABDM), has created infrastructure for interoperability (25–27). Yet, AI pilots in radiology and maternal health often remain fragmented, operating in silos. The scale challenge lies in aligning state-level health systems with federal frameworks and ensuring private sector innovations integrate with public health priorities (28, 29).
• Sub-Saharan Africa—The Pilot Graveyard: In countries such as South Africa, Kenya, and Rwanda, AI pilots for HIV, TB, and maternal health abound. Many demonstrate technical success but collapse post-pilot due to financing gaps and poor alignment with national strategies. A WHO report on tuberculosis CAD software highlights promise in autonomous AI for low-resource settings, yet long-term sustainability remains uncertain without integration into government budgets (30, 31).

### A roadmap for scaling AI in developing countries

Overcoming “pilotitis” in developing countries requires a systemic approach that directly addresses the barriers identified earlier—fragmented ecosystems, donor dependence, weak governance, limited trust, infrastructural gaps, and political complexity. The first pillar, policy alignment, demands that AI initiatives be embedded within national digital health strategies rather than remaining isolated experiments. Many pilots fail because they are conceived outside government priorities, creating misalignment during scale-up. Brazil's early challenges with integrating municipal and federal systems illustrate this: AI-enabled analytics in primary care only progressed once policymakers aligned digital health goals across levels of government. Similarly, in India, several promising radiology AI tools remained stuck in state-level silos until alignment with the Ayushman Bharat Digital Mission clarified shared data and governance expectations. This demonstrates that pilots must show not only technical accuracy but also strategic relevance to health system goals and regulatory clarity around risk, liability, and ethical oversight.

The second pillar, infrastructure and interoperability, tackles the technical bottlenecks that prevent wider deployment. Many AI pilots integrate only with vertical program databases—such as HIV or tuberculosis systems in sub-Saharan Africa—because national EHR platforms lack interoperable architecture. As a result, pilots cannot transition to other departments or states. Investing in foundational digital infrastructure, standardized data formats such as HL7-FHIR, cybersecurity measures, and cloud storage is essential for ensuring that AI tools can operate reliably across hospitals and regions. Brazil's eventual success in linking its primary and hospital systems illustrates that interoperability is a prerequisite for scaling AI-driven predictive analytics and decision support. Without this foundation, pilots remain one-off prototypes, technically impressive but functionally trapped.

The third pillar, sustainable financing, directly responds to the donor-dependency barrier. Many AI pilots collapse when grants end because governments lack budget lines to cover recurring costs such as licensing, cloud hosting, server maintenance, and specialized staff. TB-focused AI diagnostic tools in East Africa provide a stark example: despite high accuracy, several pilots shut down once donor subsidies for subscriptions and data storage expired. To avoid this cycle, financing must shift from short-term innovation grants to hybrid models combining government budgets, public-private partnerships, and value-based financing mechanisms where efficiency gains fund sustainability. Open-source AI models can further reduce costs for low-income settings, creating a financially viable pathway from pilot to scale.

An additional dimension of sustainability in the Global South is the critical importance of locally led and government-financed AI initiatives. When national or subnational governments initiate and fund AI projects, the selected interventions tend to align more closely with local health priorities such as universal health coverage, maternal and child health, and the heavy burden of tropical and neglected diseases. In contrast, donor-driven pilots—often shaped by Global North agendas—may prioritize technologies that do not correspond to domestic health system needs or resource constraints. Who initiates and finances AI projects therefore has profound implications for which problems are addressed and whether solutions are designed for long-term integration into national health strategies. Strengthening local ownership and financing is thus essential for reducing dependency on short-term external grants and ensuring that AI solutions are contextually relevant and sustainable.

The fourth pillar, capacity and workforce development, acknowledges that technological readiness is meaningless without human readiness. Clinicians frequently express concerns about loss of autonomy or unfamiliarity with algorithmic systems, resulting in resistance even when tools are accurate. In India, university students and trainees have reported low awareness of digital health infrastructures, slowing adoption of newer AI tools. Similarly, early deployments of CAD-based TB screening in African contexts revealed that lack of training undermined clinical trust, despite strong technical performance. Investing in digital literacy, AI governance training, and communities of practice can normalize AI use in daily workflows. Medical curricula should integrate foundational knowledge of AI ethics, safety, and limitations to ensure future clinicians enter the health system prepared.

The fifth pillar, trust, transparency, and participation, addresses the cultural and ethical barriers that limit adoption. Clinicians remain wary of opaque “black-box” systems, and patients often distrust algorithms developed using foreign datasets that may not reflect local epidemiology. Studies on explainability in the UK's NHS illustrate that transparency significantly improves clinician confidence; these concerns are magnified in developing countries where misdiagnosis risks are high. Building trust thus requires explainable AI interfaces, clear communication of benefits and limitations, and involvement of both clinicians and patients in co-design processes. Transparency also ensures equity: pilots must demonstrate that algorithms trained on external datasets do not perpetuate bias or worsen disparities. Participation and explanation build the social legitimacy needed for scaling.

Together, these five pillars demonstrate that the failure of AI pilots to scale is not a technical failure but a systems failure. By linking policies, infrastructure, financing, workforce preparation, and trust-building efforts, governments can transform disconnected pilots into sustainable, nationally integrated AI programs. The examples from Brazil, India, and countries such as South Africa, Kenya, and Rwanda reinforce that scaling success depends on addressing structural constraints rather than showcasing isolated technical achievements. A brief snapshot of the proposed five-pillar roadmap, and its explicit linkage to system-level barriers, is presented in Table 1.

**Table 1:** Summary of the five-pillar policy roadmap for scaling AI in healthcare in developing countries.

Roadmap pillar	Key actions	Linked barriers	Illustrative examples
Policy alignment	Integrate AI into national strategies; clarify regulation and liability	Weak governance; political fragmentation	Brazil aligning municipal and federal platforms; India integrating AI pilots with ABDM
Infrastructure & interoperability	Invest in connectivity, cloud, cybersecurity; adopt standards	Fragmented ecosystems; inadequate IT infrastructure	Brazil's integration of primary and hospital data; African HIV/TB systems operating in silos
Sustainable financing	Budget integration; public–private partnerships; open-source models	Donor dependency; high recurring costs	TB CAD tools collapsing post-donor funding in East Africa
Capacity & workforce development	Digital literacy; AI governance training; communities of practice	Low acceptance; clinician distrust	Indian medical trainees’ limited ABDM awareness; African clinicians unsure of CAD use
Trust, transparency & participation	Explainable AI; patient and clinician co-design; bias evaluation	Low trust; ethical concerns	Global evidence showing explainability improves adoption; distrust of models trained on foreign datasets

## Conclusion: moving beyond pilotitis

The promise of AI in healthcare for developing countries is undeniable. Pilots have shown that AI can enhance diagnostics, streamline workflows, and extend scarce clinical capacity. Yet the persistence of the pilot graveyard demonstrates that technical success is not enough. Scaling requires governance, financing, infrastructure, and trust.

Moving from pilot to policy means shifting focus from what AI can do to how health systems can support it sustainably. Without this shift, developing countries risk accumulating a portfolio of impressive but isolated pilot projects, with little lasting impact on population health.

By embedding AI within national strategies, investing in infrastructure, securing financing, building workforce capacity, and ensuring transparency, policymakers can transform AI from a promising experiment into a durable pillar of health systems.

Ultimately, the challenge is not to prove whether AI works, but to create health systems where AI can work responsibly, equitably, and at scale. Only then can the technology fulfill its promise to reduce disparities and strengthen healthcare delivery in the Global South.
